# COVID-19 in the Arab countries: Three-year review

**DOI:** 10.12688/f1000research.142541.1

**Published:** 2023-11-08

**Authors:** Nasar Alwahaibi, Muna Al Maskari, Samiya Al-Jaaidi, Buthaina Al Dhahli, Halima Al Issaei, Shadia Al Bahlani

**Affiliations:** 1 Department of Biomedical Science, College of Medicine and Health Sciences, Sultan Qaboos University, Muscat, Muscat Governorate, Oman; 2 Department of Biology, College of Science, Sultan Qaboos University, Muscat, Muscat Governorate, Oman

**Keywords:** Arab countries, confirmed, coronavirus, recovered, COVID-19, COVID-19 vaccines, death

## Abstract

**Background:**

Twenty-two Arab countries share a common language, history, and culture. Nevertheless, governmental policies, healthcare systems, and resources differ from one Arab country to another. We have been following Coronavirus (COVID-19) from the beginning in each Arab country. In the present study, we aimed to assess the prevalence of COVID-19 in the Arab world and to compare these findings with other significantly affected countries.

**Methods:**

Websites of the World Health Organization, World COVID-vaccinations tracker, Worldometer, and Ministries of Health were used to extract COVID-19 data in all Arab countries between the period January 2020 to December 2022.

**Results:**

All Arab countries had 14,218,042 total confirmed COVID-19 cases, 13,384,924 total recovered cases and 173,544 total related deaths. The trend demonstrated that the third quarter of 2021 recorded the highest death toll and the first quarter of 2022 recorded the highest number of confirmed and recovered cases. Compared to the top 15 affected countries, the Arab world ranked last as it had the lowest overall incidence per million population (PMP) of 31,609. The data on total deaths PMP showed that India had the lowest number of deaths with only 377 cases followed by the Arab world with 386 cases.

**Conclusions:**

Although the number of confirmed, death, and subsequently recovered cases of COVID-19 have greatly reduced in the last quarter of 2022 in most Arab countries, many Arab countries still need to re-campaign about COVID-19 vaccines and raise awareness programs about boosters. COVID-19 has had a relatively smaller impact on Arab countries than on other countries that have been significantly affected.

## Introduction

On March 11, 2020, the World Health Organization (WHO) declared Coronavirus (COVID-19) as a pandemic. COVID-19, which is caused by severe acute respiratory syndrome-coronavirus (SARS-CoV-2), is a highly contagious virus. ^1^ In the last three years, COVID-19 has infected and killed millions of people around the world, including those in Arab countries. When COVID-19 started in the first period of 2020 and the PCR tests were not available, fever, dry cough, sore throat, headache, fatigue, and breathlessness were the common symptoms for COVID-19 patients. However, many people with COVID-19 remain asymptomatic but can transmit SARS-CoV-2 to others. ^2^^,^^3^

In the past, vaccines have saved lives, avoided illness and infection, and been evaluated as effective health interventions. ^4^ In fact, WHO has shown that vaccines are safer than treatment. ^5^ COVID-19 vaccines reduce the risk of illness, hospitalization, and death from COVID-19. A study showed that the mean percentage of death with one dose was 11.55% compared to 4.31% after the second dose of any type of approved vaccine. ^6^ Globally, it has been estimated that about eight billion doses of the COVID-19 vaccine have been distributed to reduce the rate of COVID-19. ^7^ Comprehensively, the COVID-19 vaccination saved approximately 20 million lives during its first year of distribution. ^8^ As of January 1, 2023, more than 5.51 billion people worldwide have received a dose of a COVID-19 vaccine, which corresponds to approximately 71% of the world population, and fully vaccinated about 66%. ^9^

A recent study in Qatar concluded that deaths attributable to SARS-CoV-2 vaccination are extremely rare. ^10^ They reported that the death rate among the vaccinated persons with a high probability of relationship to SARS-CoV-2 vaccination was 0.34 per 100,000 vaccine recipients, while the death rate among the vaccinated persons with either high or intermediate probability of relationship to SARS-CoV-2 vaccination was 0.98 per 100,000. In line with this study, the United States Centers for Disease Control and Prevention identified only nine deaths from 14,980 reports of death among more than 589 million vaccine doses between December 14, 2020, and June 6, 2022. ^11^

On the other hand, WHO has identified many different variants of the coronavirus SARS-CoV-2 including Alpha, Beta, Omicron, Gamma, Delta, Eta, Iota, Kappa, Zeta, and Mu. On 26 November 2021, WHO declared the Omicron variant, known as lineage B.1.1.529, a variant of concern. ^12^ It was first identified in Botswana and South Africa. ^13^Although the Omicron variant (B.1.1.529) causes less severe symptoms, it is more contagious and spreads faster than any previous variant. Many studies reported that this variant causes reinfection, and may escape the immune system’s defenses, and two doses of vaccination appeared to be less effective. ^14^^–^^16^ However, some studies show that boosters can provide protection against Omicron infection. ^17^^–^^19^ Unfortunately, these variants and probably others will continue to emerge as long as the coronavirus SARS-CoV-2 remains.

The development of vaccines is usually a lengthy and complex process. However, in order to stop the transmission of COVID-19, vaccine development has been accelerated. ^20^^,^^21^ Despite unequal vaccine distribution, vaccine hesitancy, and waning immunity, billions of vaccine doses have been administered worldwide. One of the major causes of vaccine hesitancy and delay in vaccination is the concern about adverse effects. ^22^ As of December 29, 2022, WHO approved 11 COVID-19 vaccines including Sinopharm, Sinovac, Bharat Biotech, Moderna, Pfizer/BioNTech, Oxford/AstraZeneca, Serum Institute of India, Janssen/Johnson & Johnson, Novavax, Serum Institute of India, and CanSino. ^23^

The Arab world contains 22 countries, distributed 12 in Asia and 10 in Africa. Language, history, traditions, and culture are shared by Arab countries. ^24^ However, the healthcare systems and availability of resources differ from one Arab country to another. Previously, we published two review papers. The first was a 5-month COVID-19 data in all Arab countries from January 1, 2020, to May 31, 2020, and concluded that most Arab countries took some serious early steps to minimize the outbreak of COVID-19. ^25^ The second one was a one-year from February 2020 to February 2021, and we concluded that among the Arab countries, Qatar, Bahrain, and Lebanon showed the highest number of recovered, confirmed, and deaths per million population, respectively. The number of confirmed and death cases among Arab countries triggers significant worries about morbidity and mortality related to COVID-19, respectively. ^26^ We have been following COVID-19 from the beginning in each Arab country. In the present study, we aimed to assess further the prevalence of COVID-19 in the Arab world from January 2020 to December 2022 and to compare these findings with other significantly affected countries.

## Methods

We used the WHO, World COVID-vaccinations tracker, Worldometer, and Ministries of Health official websites to search for COVID-19 data in Algeria, Bahrain, Comoros, Djibouti, Egypt, Iraq, Jordan, Kuwait, Lebanon, Libya, Mauritania, Morocco, Oman, Palestine, Qatar, Saudi Arabia (SA), Somalia, Sudan, Syria, Tunisia, United Arab Emirates (UAE), and Yemen. The period covered was from January 2020 to December 2022. The inclusion criterion was official information about clinically diagnosed COVID-19 in English or Arabic. The exclusion criterion was unofficial information regarding COVID-19 in all Arab countries, unspecified date and location of information, or suspicion of duplicate information. The data were collected monthly and verified with the data in the Worldometer. The following information was collected from each Arab country: total population, median age, number of monthly confirmed, death, and recovered cases, the total number of COVID-19 tests, and COVID-19 vaccine rates (first and second). The data for the topmost 15 affected countries were extracted from the Worldometer at the same time as the data for the Arab countries. Ethical approval and written informed consent were not required for this type of study. Data were analyzed using the IBM SPSS Statistics (RRID:SCR_016479) software version 25 (SPSS Inc., Chicago, IL, USA). Results are presented as numbers, percentages, and means.

## Results

By January 01, 2023, the total Arab population who live in Arab countries was 449,809,846. Egypt recorded the highest population among all Arab countries, followed by Sudan, and Algeria with 106,156,692, 45,992,020, and 45,350,148, respectively. The highest median age was seen in UAE, followed by Qatar, and Bahrain with 38.4, 33.7, and 32.3 years, respectively. Whereas the lowest median age was seen in Sudan at 18.3 years. All Arab countries utilized real-time polymerase chain reaction (RT-PCR) as the testing method for SARS-CoV-2. The total number of COVID-19 tests in all Arab countries was 362,542,626. UAE (19,632,329) recorded then the highest number of tests per million population (PMP), followed by Bahrain (5,960,320), and then Oman (4,695,724). The people in Qatar and the United Arab Emirates have received two doses of the COVID-19 vaccine equally to about 99% whereas those in Yemen received only 3% for both doses ( Table 1).

**Table 1.** Features of the Arab countries related to COVID-19 from January 2020 to December 2022.

Countries	Population	Median age, years	Total tests	Tests PMP	One dose of COVID-19 vaccine in %	Two doses of COVID-19 vaccine in %
Egypt	106,156,692	23.9	3,693,367	34,792	54	40
Sudan	45,992,020	18.3	562,941	12,240	25	19
Algeria	45,350,148	28.9	230,861	5,091	18	15
Iraq	42,164,945	20	19,448,292	461,243	29	20
Morocco	37,772,756	29.3	12,856,284	340,359	69	64
SA	35,844,909	30.8	44,940,564	1,253,750	79	74
Yemen	31,154,867	19.8	329,592	10,579	3	3
Syria	19,364,809	24.3	146,269	7,553	19	13
Somalia	16,841,795	18.5	400,466	23,778	49	41
Tunisia	12,046,656	31.6	4,983,949	413,721	62	55
Lebanon	10,300,869	30.5	4,795,578	717,380	40	35
UAE	10,081,785	38.4	197,928,922	19,632,329	99	99
Libya	7,040,745	28.9	2,483,446	352,725	34	18
Jordan	6,684,849	22.5	17,201,885	1,669,945	48	45
Palestine	5,345,541	21.1	3,078,533	575,907	43	38
Oman	5,323,993	25.6	25,000,000	4,695,724	65	61
Kuwait	4,380,326	29.3	8,447,300	1,928,464	82	79
Qatar	2,979,915	33.7	4,065,369	1,364,257	99	99
Bahrain	1,783,983	32.3	10,633,110	5,960,320	76	75
Mauritania	1,274,727	20.5	1,009,957	206,030	46	34
Djibouti	1,016,097	23.9	305,941	301,094	33	31
Comoros	907,419	19.9	NA	NA	52	47
Total/Average	449,809,846	26	362,542,626	1,903,204		

COVID-19: Coronavirus. SA: Saudi Arabia. UAE: United Arab Emirates. PMP: per million population. Countries ranked based on the largest population.

The total number of COVID-19 cases in all Arab countries was 14,218,042 and of those, 173,544 (1.2 %) were deceased and 13,384,924 (94%) were recovered. Jordan, Qatar, Kuwait, Palestine, and Lebanon recorded the highest number of reported cases PMP with 261,404, 164,032, 151,138, 131,568, and 118,957, respectively. Yemen recorded only 383 cases PMP. Based on the evaluation of three years from January 2020 to December 2022, the trend showed that the first quarter of 2022 had the highest number of confirmed COVID-19 cases in all Arab countries with 3,235,665 cases. In the same quarter period, 11 Arab countries scored their highest number of COVID-19 confirmed cases ( Table 2).

**Table 2.** The trend of quarterly COVID-19 confirmed cases in all Arab countries from January 2020 to December 2022.

Countries	Q1-20	Q2-20	Q3-20	Q4-20	Q1-21	Q2-21	Q3-21	Q4-21	Q1-22	Q2-22	Q3-22	Q4-22	Total	PMP
Jordan	268	864	10,693	282,669	323,565	133,345	73,293	238,708	630,811	4,368	48,866	0	1,747,450	261,404
Qatar	781	95,307	29,672	18,074	36,130	42,107	14,572	13,885	110,444	20,962	69,138	37,730	488,802	164,032
Kuwait	289	45,906	58,989	45,404	81,619	124,474	54,539	5,455	213,137	15,012	12,985	4,227	662,036	151,138
Palestine	117	2,490	37,471	98,105	135,048	69,898	93,879	33,514	185,519	3,812	42,915	536	703,304	131,568
Lebanon	470	1,308	37,856	141,869	286,897	76,466	80,579	102,485	364579	19,690	103,726	9,444	1,225,369	118,957
UAE	669	47,998	45,523	112,561	253,623	170,330	103,085	25,945	129372	54,500	81,702	19,506	1,044,814	103,633
Tunisia	393	779	16,233	119,811	118,092	164,795	287,880	19,860	308041	16,296	93,506	1,885	1,147,571	95,260
Oman	192	39,878	58,515	30,282	30,351	109,327	34,511	1,720	81808	2,385	7,265	656	396,890	74,547
Libya	8	816	33,701	65,410	60,045	33,494	147,617	47,643	113004	400	4,856	148	507,142	72,029
Iraq	630	48,479	313,872	232,310	255,633	494,980	665,730	82,106	226168	28,754	111,320	6,627	2,466,609	58,499
Mauritania	6	4,143	3,325	6,168	4,205	2,961	15,306	5,753	16802	1,145	3,009	602	63,425	49,755
Morocco	602	11,783	108,798	316,149	59,344	34,685	403,467	30,592	197794	53,695	48,052	6,634	1,271,595	33,664
SA	1563	189,260	143,782	28,136	27,266	97,585	59,605	8,102	194578	44,282	22,233	10,615	827,007	23,071
Djibouti	31	4,651	734	408	2,356	3,422	1,279	787	1917	105	0	0	15,690	15,441
Comoros	0	303	171	291	2,939	239	204	2,541	1398	14	371	511	8,982	9,898
Algeria	716	13,191	37,306	48,098	17,993	22,322	64,031	15,161	46853	416	4,589	552	271,228	5,980
Egypt	711	67,600	34,768	33,565	66,199	78,439	24,748	80,328	118906	10,381	0	0	515,645	4,857
Syria	10	269	3,921	7,234	7,605	6,476	9,405	15,358	5428	221	1,380	169	57,476	2,968
Somalia	5	2,919	664	1,126	6,785	3,447	5,034	3,552	2878	393	411	86	27,300	1,620
Sudan	7	9,251	4,382	11,860	4,611	6,547	1,670	8,190	15437	669	661	401	63,686	1,384
Bahrain	567	25,980	44,106	21,811	51,770	121,382	9,230	7,005	269111	70,383	54,916	17,811	694,072	389
Yemen	0	1,158	876	68	2,429	2,389	2,219	987	1680	18	111	14	11,949	383
Total	8035	614,333	1025,358	1621,409	1834,505	1,799,110	2,151,883	749,677	3235665	347,901	712,012	118,154	14,218,042	

COVID-19: Coronavirus. Q: Quarter. SA: Saudi Arabia. UAE: United Arab Emirates. PMP: per million population. Countries ranked based on the highest number of confirmed cases PMP.

Deaths PMP were dominant in Tunisia, Jordan, Palestine, Lebanon, and Libya with 2,430, 2,116, 1,073, 1,052, and 914, respectively. The trend demonstrated that the third quarter of 2021 had the highest number of deaths in all Arab countries with 31,275 cases. In the same quarter period, five countries had the highest number of COVID-19-related deaths ( Table 3).

**Table 3.** The trend of quarterly COVID-19 death cases in all Arab countries from January 2020 to December 2022.

Countries	Q1-20	Q2-20	Q3-20	Q4-20	Q1-21	Q2-21	Q3-21	Q4-21	Q1-22	Q2-22	Q3-22	Q4-22	Total	PMP
Tunisia	10	40	196	4,374	4,223	6,116	9,962	655	2,747	368	558	35	29,284	2,430
Jordan	5	4	68	3,773	3,090	2,810	977	1,926	1,395	20	69	12	14,149	2,116
Palestine	1	7	303	1,089	1,501	932	579	522	721	5	68	8	5,736	1,073
Lebanon	12	22	363	1,221	4,616	1,617	490	778	1,187	159	253	119	10,837	1,052
Libya	0	24	527	893	1,236	513	1,471	1,046	709	11	7	0	6,437	914
Bahrain	4	83	164	101	169	831	37	5	75	21	28	18	1,536	861
Oman	1	175	759	564	179	1,411	974	20	136	10	0	0	4,229	794
Mauritania	0	126	35	181	107	40	288	93	112	1	12	2	997	782
Iraq	46	1,897	7,238	3,632	1,510	2,863	5,206	1,766	1,009	74	133	27	25,401	602
Kuwait	0	354	256	324	379	645	477	19	87	1	5	7	2,554	583
Morocco	36	189	1,927	5,203	1,470	471	5,019	536	1,209	49	169	16	16,294	431
SA	10	1,639	3,119	1,455	446	1,150	897	161	170	164	146	163	9,520	265
UAE	6	309	104	250	828	314	286	67	138	14	29	3	2,348	233
Egypt	46	2,907	2,961	1,662	4,465	4,128	1,230	4,369	2,649	196	0	0	24,613	232
Qatar	2	111	101	31	46	299	16	13	57	2	3	3	684	229
Djibouti	0	54	7	0	10	84	14	20	0	0	0	0	189	186
Comoros	0	7	0	2	137	0	1	10	3	0	1	0	161	177
Syria	2	7	191	511	563	602	389	632	243	10	26	0	3,176	164
Algeria	58	854	807	1,032	345	620	2,103	465	590	1	4	2	6,881	152
Sudan	2	570	264	725	502	691	150	427	1,576	44	10	33	4,994	108
Somalia	0	90	9	31	407	238	336	222	17	0	2	9	1,361	81
Yemen	0	312	275	23	296	455	373	250	159	7	9	4	2,163	69
Total	241	9,781	19,674	27,077	26,525	26,830	31,275	14,002	14,989	1,157	1,532	461	173,544	

COVID-19: Coronavirus. Q: Quarter. SA: Saudi Arabia. UAE: United Arab Emirates. PMP: per million population. Countries ranked based on the highest number of deaths PMP.

Bahrain, Jordan, Kuwait, Qatar, and Palestine showed the highest number of recovered cases PMP with 307,175, 258,944, 147,542, 134,455, and 131,528, respectively. The trend showed that the first quarter of 2022 had the highest number of recovered cases in all Arab countries with 3,261,712 cases. In the same quarter period, 10 countries had the highest number of recovered cases ( Table 4).

**Table 4.** The trend of quarterly COVID-19 recovered cases in all Arab countries from January 2020 to December 2022.

Countries	Q1-20	Q2-20	Q3-20	Q4-20	Q1-21	Q2-21	Q3-21	Q4-21	Q1-22	Q2-22	Q3-22	Q4-22	Total	PMP
Bahrain	NA	14,685	NA	NA	20,226	125,972	5,933	4,065	267,768	65,995	43,351	NA	547,995	307,175
Jordan	26	856	3,744	265,925	249,947	214,596	66,432	217,370	660,045	4,805	47,261	0	1,731,007	258,944
Kuwait	73	36,957	59,756	49,831	70,573	118,640	70,978	3,098	213,361	11,229	11,788	NA	646,284	147,542
Qatar	62	81,502	41,135	18,723	22,699	55,678	14,963	10,480	114,060	17,280	24,083	NA	400,665	134,455
Palestine	18	607	40,603	91,590	112,106	91,952	71,379	53,346	187,621	3,622	37,821	12,426	703,091	131,528
Lebanon	35	1,148	15,927	151,201	202,376	161,850	63,531	63,380	402,783	25,356	0	0	1,087,587	105,582
UAE	61	37,505	46,154	100,718	260,913	166,087	114,804	16,520	122,607	58,187	80,882	22,927	1,027,365	101,903
Tunisia	1	1,028	1,512	104,639	110,748	136,513	323,184	18,861	312,348	0	0	0	1,008,834	83,744
Oman	34	23,391	64,704	33,362	22,480	88,015	62,971	1,284	82,558	1,730	NA	NA	380,529	71,474
Libya	0	209	19,152	52,746	75,757	30,523	82,498	115,393	113,774	921	9,537	151	500,661	71,109
Iraq	152	24,608	267,437	245,644	226,255	481,432	680,125	139,317	217,997	25,792	124,945	7,436	2,441,140	57,895
Mauritania	2	1,620	5,469	4,289	5,713	2,697	14,976	4,464	18,456	620	3,486	631	62,423	48,969
Morocco	24	8,815	91,414	307,251	76,207	33,865	391,322	32,110	206,249	28,105	73,192	6,294	1,254,848	33,221
SA	165	130,601	187,165	34,650	24,230	113,424	68,185	5,173	175,193	39,662	23,509	6,419	808,376	22,552
Djibouti	2	4,522	820	384	810	4,905	758	1,183	2,006	37	0	0	15,427	15,182
Comoros	0	200	253	363	2,720	229	201	832	3,121	14	372	514	8,819	9,718
Egypt	157	18,303	77,634	16,452	42,470	56,368	46,868	62,880	115,379	5,671	0	0	442,182	4,165
Algeria	46	9,851	26,065	31,165	14,505	15,457	42,435	10,784	28,050	179	3,776	335	182,648	4,027
Syria	0	105	998	4,193	7,559	8,970	2,202	8,487	14,816	5,428	1,341	249	54,348	2,806
Sudan	0	4,014	2,750	6,772	10,678	6,302	1,599	6,599	1,615	0	16,961	889	58,179	1,265
Somalia	0	932	2,014	666	1,249	2,385	2,277	3,425	234	0	0	0	13,182	782
Yemen	0	488	798	108	288	2,386	1,591	1,363	1,671	513	123	5	9,334	299
Total	858	401,947	955,504	1,520,672	1,560,509	1,918,246	2,129,212	780,414	3,261,712	295,146	502,428	58,276	13,384,924	

COVID-19: Coronavirus. Q: Quarter. SA: Saudi Arabia. UAE: United Arab Emirates. PMP: per million population. Countries ranked based on the highest number of recovered cases PMP.

In comparison to the topmost 15 affected countries, the Arab world ranked 16 as it had the lowest overall incidence PMP of 31,609. The data on total deaths PMP showed that India had the lowest number of deaths with only 377 cases followed by the Arab world with 386 cases. The United States recorded the highest number of deaths with 3,339 cases. In terms of the total number of tests for SARS-CoV-2, Arab countries ranked eleventh with 805,990 tests PMP. The highest number of tests PMP was conducted by Spain (10,082,298) and the lowest was by Brazil (296,146). Arab countries showed the youngest median age followed by India with 28.7 years as its median age. Conversely, Japan had the oldest median age of 48.6 years, however, it had fewer COVID-19 deaths per million populations with 456 cases. It is worth mentioning that six of the top 15 affected countries are from Europe. France was the worst country (after South Korea) as it recorded 599,471 cases PMP and ranked first. Other parameters, which include total tests and population, are also compared ( Table 5).

**Table 5.** Comparison of COVID-19 prevalence between the Arab world and the top 15 affected countries from January 2020 to December 2022.

	Country	Total Confirmed cases	Total Death cases	Total Cured cases	Confirmed cases PMP	Deaths PMP	Total Tests	Tests PMP	Population	Median Age, years
1.	France	39,316,017	161,962	38,342,881	599,471	2,470	271,490,188	4,139,547	65,584,518	41.7
2.	S. Korea	29,116,800	32,219	27,893,416	567,248	628	15,804,065	307,892	51,329,899	43.2
3.	Germany	37,369,865	161,465	36,615,400	445,497	1,925	122,332,384	1,458,359	83,883,596	47.8
4.	Australia	11,131,707	17,052	10,979,282	427,013	654	78,835,048	3,024,116	26,068,792	44.5
5.	Italy	25,143,705	184,642	24,541,402	417,234	3,064	262,558,741	4,356,898	60,262,770	46.5
6.	UK	24,135,084	198,937	23,844,243	352,348	2,904	522,526,476	7,628,357	68,497,907	40.6
7.	USA	102,513,690	1,117,983	99,513,507	306,189	3,339	1,152,003,631	3,440,817	334,805,269	38.5
8.	Spain	13,684,258	117,095	13,486,683	292,905	2,506	471,036,328	10,082,298	46,719,142	43.9
9.	Japan	29,212,535	57,266	21,105,754	232,612	456	86,237,109	686,684	125,584,838	48.6
10.	Argentina	9,891,139	130,124	9,609,732	214,977	2,828	35,716,069	776,264	46,010,234	32.4
11.	Turkey	17,042,722	101,492	N/A	199,186	1,186	162,743,369	1,902,052	85,561,976	32.2
12.	Brazil	36,354,255	693,941	34,938,186	168,812	3,222	63,776,166	296,146	215,353,593	33.2
13.	Russia	21,798,509	393,712	21,207,802	149,504	2,700	273,400,000	1,875,095	145,805,947	40.3
14.	Vietnam	11,525,231	43,186	10,611,275	116,471	436	85,826,548	867,342	98,953,541	31.9
15.	India	44,679,564	530,702	44,144,029	31,764	377	910,365,101	647,195	1,406,631,776	28.7
16.	Arab World	14,218,042	173,544	13,384,924	31,609	386	362,542,626	805,990	449,809,846	26.0

COVID-19: Coronavirus. PMP: per million population. Countries ranked based on the highest number of confirmed cases PMP.

## Discussion

The present study aimed to assess the prevalence and the impact of the COVID-19 pandemic in 22 Arab countries and compare them with other significantly affected countries from January 2020 to December 2022. COVID-19 confirmed cases increased exponentially in the first quarter of 2021 due to the New Year season with Jordan demonstrating the highest number of cases followed by Lebanon. A dramatic increase in COVID-19 confirmed cases occurred in the third quarter of 2021 due to the emergence of the Delta variant, ^27^ which peaked in Iraq with a total number of 665,730. Another reason for the spike increase is the slow uptake of the vaccine in many countries. ^28^ For example, only 20% of the population in Iraq had received single/double doses of the COVID-19 vaccine. In addition, people in several countries, such as Iraq, refused to take the vaccine, which in turn, might have played a role in accelerating the number of COVID-19 confirmed cases. ^29^ Furthermore, the re-opening of schools and businesses, people returning from holidays and social mixing subsidized the escalation in the COVID-19 confirmed cases at the end of summer and the start of winter of 2021. ^27^ The situation was further aggravated by the economic disturbance in some of these countries hence, they had a compound crisis; COVID-19 and economic disruption. ^30^

Preventive measures taken by countries during the pandemic affected the spread of the COVID-19 virus. During the first, second, and third quarters of 2020, the pandemic was under control in most Arab countries due to the implementation of extreme precautionary measures. The major measures include land, sea, and air route closure, nighttime or all-day curfew and lockdown, school and universities closure, worship places closure, prohibition of gatherings, closure of shops, malls, beaches, public parks, and gardens, cancellation of all cultural and sports events, festivals, seminars, and scientific meetings, and suspension of work in all government and private sectors. ^31^^–^^33^A study conducted in Saudi Arabia showed that preventive measures had an enormous effect in reducing the number of expected confirmed cases of COVID-19 from 437,096 cases to an observed number of 28,656 at the beginning of the second quarter of 2020. ^34^ Because of the economic crisis in some countries like Jordan, some of these measures were diminished in the fourth quarter of 2020, leading to an increase in COVID-19 cases. ^35^

The first quarter of 2022 was a shockwave as it was recorded with the highest-ever number of COVID-19 confirmed cases with 3,235,665 peaked by Jordan with a total number of 630,811. The potential reason for the spike is the occurrence of the Omicron variant that had affected the death rate and increased hospitalization. ^36^ On the other hand, the pandemic had intense consequences on the economy of many countries. Therefore, to revive the economy, different approaches were taken such as the period of isolation of the infected people was reduced and guidelines on PCR testing of suspected COVID-19 cases became more restrictive. ^35^ Subsequently, there was a dramatic decrease in the confirmed cases in the second quarter of 2022, which continued to decline until the end of the year. Both the third-fourth quarters of 2021 and 2022 demonstrated a similar wave but with a lesser rate of confirmed COVID-19 cases in 2022. This could be due to the higher vaccine coverage in that year emphasizing the importance and the impact of vaccination intake. ^37^ Despite the full vaccination coverage in Qatar (99%), Qatar was reported with the highest number of confirmed cases of COVID-19 in the fourth quarter of 2022 with a total number of 37,730. The conceivable reason could be that Qatar was hosting the World Cup 2022 with no COVID-19 restrictive measures required to enter the country, which had strikingly increased the social gathering and thus, increased COVID-19 confirmed rates. ^38^

Among Arab countries, such as the Gulf Cooperation Council (Bahrain, Kuwait, Oman, Qatar, Saudi Arabia, and UAE) and Morocco, with over 60% of their population fully vaccinated, showed a sharp decrease in the death rate PMP in the last quarter of 2021 ( Table 3). However, the first quarter of 2022 retained a death peak, which disappeared by the second quarter of the same year, probably because of the fast-spreading Omicron variant wave. Countries like Yemen, Syria, Iraq, and Sudan showed a low percentage of vaccination, a low number of tests, and surprisingly low deaths PMP ( Tables 1 and 3). Political instability and a weak healthcare system that depends on outside humanitarian aid could be attributed to a lack of regular testing and continued poor documentation of COVID-19 status. ^25^

Jordan, as an example of an Asian country, experienced the first wave of viral spread during the fourth quarter of 2020, lasted until January 2021, and resulted in a great increase in accumulative confirmed and death cases. Before the first wave started, epidemiological status was under control, but by early September 2020 some restrictions were removed, *e.g.*, land boundaries were opened for export/import goods from neighboring countries, universities opened doors for registration, and people started to take the crisis less seriously. ^35^ The second wave in Jordan was led by the fast-spreading UK variant that started in January 2021 continued until May 2021 and peaked in March 2021. The Indian Delta strain spread in Jordan during the second quarter and beginning of the third quarter of 2021, but the epidemiological data did not show any peak due to vaccination or naturally acquired immunity. ^35^ The third and fourth waves, from October 2021 until January 2022 and January 2022 to March 2022, respectively, started as the government halted many preventive measures due to economic stress. Both waves were led by the Omicron variant. ^35^

Tunisia, as an example of an African country, started to record positive cases in early March 2020, however by June 2020, the government could control the situation by applying preventive measures, which resulted in zero cases between 4 ^th^ and 12 ^th^ June. In July 2020, borders opened and clear slackening in sticking with preventive measures by people resulted in the second wave, which affected the country. March 2021 was the start of the third wave in Tunisia, which was highlighted by severe cases and a high transmission rate, caused by the Alpha variant after the fourth wave, which was led by the Delta variant that occurred in May 2021. During this period, Tunisia got a high rate of deaths PMP ( Table 3). The Omicron variant of concern was the cause of the fifth wave by the end of 2021, so the country experienced another peak of death during the first quarter of 2022. These waves attributed to a large number of deaths. Tunisia has the highest number of deaths PMP among Arab countries. The economic crisis in addition to political problems made it difficult for the government to control COVID-19 and this in turn, resulted in the delay of the introduction and dissemination of COVID-19 vaccines. ^39^

Studying the trend of recovered cases is a useful indication of the health status and the health system in these countries. It is also a good tool to be used in terms of applying certain restrictions such as the duration of lockdown. From January 2020 to December 2022, all Arab countries showed various numbers of recovered cases of COVID-19. Such variation depended initially on the number of infected cases and on a broader scope, the severity of infection, treatment, patient immunity, vaccination, and other political and health factors. ^37^ Although some data were missing on the number of recovered cases for some countries, Bahrain, followed by Jordan, Kuwait, and Qatar showed the highest among all Arab countries. Whereas, Syria, Sudan, Somalia, and Yemen showed the lowest number of recovered cases, which may be explained by the unstable political status and subsequently the weakness of the healthcare system. Over time and based on the registered cases, the trend of recovered cases started at a low, reached a high peak, and eventually declined. This trend is common in such pandemics and the mode of spreading such infection had been observed in the previous pandemic. ^40^ The bell-shaped trend in certain countries such as Palestine, given all collected data, are accurate, and helped tremendously in managing the pandemic crisis in terms of lockdown, financial and social impact, and predicting the coming waves of the mutant viruses.

The effect of a complete vaccination regimen on the recovered cases was not consistent among Arab countries. As mentioned previously, Qatar had the highest percentage of the vaccinated population but was not the top-rated country in the recovered cases. Such observation does not exclude the importance of vaccination effect on these cases rather than additional factors that might have contributed to this outcome.

A recent study showed the effectiveness of the vaccine in preventing SARS-CoV-2 infection and its symptoms. In addition to other mitigation strategies, vaccine campaigns could have a great impact on the number of confirmed and recovered cases. ^41^ Although quarantine was one of the most important measures in controlling the spread of the epidemic, ^42^ such a theory changed once the right vaccine was used. Quarantine controls the disease by the large fraction of pre-symptomatic and asymptomatic transmission, unlike the vaccine that eliminates the virus and reduces its symptoms in many cases.

During the recent pandemic, vaccination campaigns have proven their effectiveness to control the disease and reduce the severity of its symptoms. The pharmaceutical companies were competing to prepare the most effective vaccine with less side effects. The focus of vaccination campaigns should be a priority in any coming pandemic and their importance should be stressed to societies. Unfortunately, many countries had anti-vaccine groups that were affecting the vaccination campaigns badly. Those groups influenced the decision of several people on taking the vaccines and subsequently affecting the control of the disease. Changing the culture and the mentality of certain groups in societies will be the first and biggest challenges for vaccination campaigns in any pandemic in the future. Altogether, the authors hypothesized that vaccination campaigns influenced the number of confirmed and recovered cases in these Arab countries despite the impact of other related factors. It is very important to open new insights in the research of vaccine discovery and more time and effort should be spent in this area.

Recently, Hoxha and coworkers analyzed COVID-19 data from 164 different countries and concluded that higher COVID-19 vaccination rates are associated with lower COVID-19 mortality rates and that there is a tendency for more vaccinations and fewer deaths per 1,000 cases with increasing country income levels. ^43^ Notably, Both Qatar and UAE represent as the highest income countries in the Arab world. UAE recorded the same percentage of the vaccinated population as Qatar, and both demonstrated low number of deaths.

Compared with the 15 topmost affected countries in the world, the Arab world experienced a lower number of cases and deaths PMP ( Table 5). It also performed fewer tests than its population. South Korea, Japan, Argentina, Brazil, Vietnam, and India also performed a lesser number of tests than their populations. Conversely, the six European countries (France, Germany, Italy, UK, Spain, and Russia), Australia, USA, and Turkey have performed tests more than their populations. A similar result was observed for most European countries by November 2022, where the number of tests exceeded the number of residents. ^37^ The diagnostic testing strategy and mass screening including the screening of asymptomatic people is a major strategy in controlling the spread of the virus. ^44^ Hence, testing procedures such as PCR is a tool used to detect and record both confirmed and death cases. ^45^ It is possible that such countries that performed fewer tests than their populations could have resulted in recording fewer confirmed cases and deaths. Furthermore, it has been suspected that the smaller number of tests carried out could be a reason for the reduced spread of the virus and the slowing down of the spread of the infection. However, the analysis conducted by Hisaka *et al.*, (2020) concluded that extensive PCR testing might be effective in reducing the number of deaths and that further studies are required to verify this hypothesis. ^46^

A previous study reported that older age plays a vital role in influencing the severity of COVID-19 disease and negative clinical outcomes than the younger population. ^47^ With this, the lower deaths PMP as observed in both the Arab world (386 cases) and India (377 cases) could be attributed to the low median age of 26 and 28.7 years, respectively. By contrast, this claim contradicts why Japan with the highest median age of 48.6 years in the top 15 affected countries also recorded a low number of deaths PMP (456 cases).

Studies have reported that in response to the COVID-19 pandemic, all 44 Muslim countries including the Arab world and Turkey, mainly implemented mitigation strategies to control the virus. ^48^^,^^49^ The main aim of implementing a mitigation strategy is to reduce the number of death tolls by focusing on the medical care of severe cases and relying on social distancing and quarantine to flatten the curve of epidemic impact and burden on hospitals. ^50^ Stringent measures included the suspension of all airline flights, cancellation of Umrah, and down-scaling of the pilgrimage to Mecca. ^48^ Other countries that mainly responded with mitigation strategies included the United States, and European countries. ^50^^,^^51^ Mitigation measures are adopted immediately once the containment strategies (strict lockdowns) fail to isolate the infected individuals due to the widespread infection in the community or until vaccines are developed. ^52^ Hence, there is a clear indication that countries vary widely in their response to the COVID-19 impact and that these differences could be partially explained by many factors such as the economic and cultural situation, governmental policies, medical capacities, the age and genetic variation between ethnic groups in a population. ^46^

### Strengths and limitations

A key strength of this study is its comprehensive follow-up and collection of COVID-19 data in each Arab country for three consecutive years. In addition, accurate monthly data were obtained from the Ministry of Health in each country and verified with the Worldometer data for COVID-19. However, there are a few limitations in our review. First, no distinction was made between Arabs and non-Arabs in the reported health data since many non-Arabs work in Arab countries. Second, COVID-19 hospitalizations are lower after being fully vaccinated, so many patients might not be included in these statistics. Third, many Arab countries lack information about COVID-19 vaccine boosters. Finally, this study could not identify an association between COVID-19 related deaths and comorbidities due to the absence of risk factors such as hypertension, diabetes, respiratory system disease, and cardiovascular disease in the data extracted.

## Conclusions

Although the number of confirmed, death, and subsequently recovered cases of COVID-19 have greatly reduced in the last quarter of 2022 in most Arab countries, further efforts to address the need to re-campaign on COVID-19 vaccines and raise awareness programs about boosters must be implemented. COVID-19 has had a relatively smaller impact on Arab countries than on other countries that have been significantly affected.

## Data Availability

No data are associated with this article.
