# Supplementary material for: COVID-19 in the Arab countries: Three-year study
Source: F1000Res. 2024 May 29;12:1448. Originally published 2023 Nov 8. [Version 3] doi: 10.12688/f1000research.142541.3 (PMC11157493; doi:10.12688/f1000research.142541.3)
Supplement: Peer review history

# Reviewer report: Reviewer response for version 3

**DOI:** 10.5256/f1000research.166903.r284091

**Published:** 2024-06-04

**Authors:** Ahmad A. Alrasheedi

**Affiliations:** 1 Department of Family and Community Medicine, College of Medicine, Qassim University, Buraydah, Al Qassim Region, Saudi Arabia

The authors have addressed the concerns and made the necessary amendments (we suggested) to this version. I think this study can be indexed.

Is the review written in accessible language?

Yes

Are all factual statements correct and adequately supported by citations?

Yes

Are the conclusions drawn appropriate in the context of the current research literature?

Yes

Is the topic of the review discussed comprehensively in the context of the current literature?

Yes

Reviewer Expertise:

Preventive medicine, COVID-19, DM, and critical studies.

I confirm that I have read this submission and believe that I have an appropriate level of expertise to confirm that it is of an acceptable scientific standard.

# Reviewer report: Reviewer response for version 1

**DOI:** 10.5256/f1000research.156102.r223543

**Published:** 2024-05-11

**Authors:** Yasser Amer

**Affiliations:** 1 King Saud University Medical City, Riyadh, Saudi Arabia

This study represents a comprehensive and well-conducted follow-up and collection of COVID-19 data in 22 Arab countries for three consecutive years.

 The authors obtained accurate monthly data from the official websites of the Ministry of Health in each country in addition to verifying those against the data from the websites of the WHO, World COVID-vaccinations tracker, and Worldometer for COVID-19.

 The abstract, introduction, and methods were well written, clear, and appropriate to the research topic.

 I would like to suggest enriching the discussion with this subtitle: "Implications for clinical practice and public health practice".

 Here are some elements for the respected authors to consider as relevant to the study aim:-

1. **Strengthen Healthcare Infrastructure**: Examples 1: Invest in healthcare infrastructure, including hospitals, clinics, and medical supplies, to ensure preparedness for future pandemics. Example 2: Enhance the capacity of healthcare systems to accommodate a surge in patients during emergencies.
2. **Improve Disease Surveillance**: Example 1: Develop and maintain robust surveillance systems to detect and monitor infectious diseases promptly. Example 2: Enhance the sharing of data and information among Arab countries and with international health organizations.
3. **Promote Vaccination Campaigns**: Example 1: Implement comprehensive and equitable vaccination campaigns to achieve herd immunity against COVID-19 and future infectious diseases. Example 2: Combat vaccine hesitancy through targeted public health messaging and education.
4. **Enhance Telemedicine and Digital Health**: Example 1: Expand telemedicine and digital health services to improve access to healthcare and reduce the burden on in-person facilities. Example 2: Ensure the privacy and security of electronic health records and telemedicine platforms.
5. **Prepare for Variants**: Example 1: Develop strategies to monitor and respond to emerging variants of concern, including the rapid adjustment of vaccines and therapeutics.Example 2: Invest in genomic sequencing capabilities for timely variant detection.
6. **Strengthen Public Health Messaging**: Example 1: Enhance public health communication strategies to provide accurate and timely information to the public during health crises. Example 2: Address misinformation and disinformation through targeted campaigns.
7. **Invest in Research and Development**: Example 1: Allocate resources for research into infectious diseases, epidemiology, and vaccine development to better prepare for future pandemics. Example 2: Foster collaboration between academia, industry, and public health agencies.
8. **Prepare for Healthcare Workforce Challenges**: Example 1: Develop contingency plans for healthcare workforce shortages during health emergencies. Example 2: Support the mental health and well-being of healthcare workers.
9. **Improve International Collaboration**: Example: Foster regional and international collaboration in disease surveillance, response, and research. Example 2: Participate in global initiatives to ensure equitable access to vaccines and treatments.
10. **Health Equity and Vulnerable Populations**: Example 1: Prioritize health equity in policies and interventions, addressing disparities that affect vulnerable populations. Example 2: Ensure access to healthcare and social support for marginalized communities.

The following 4 articles below may be helpful in writing up or re-writing these points, if you agree on them, or additional points to be additional take-home messages for the readers.

 These are 2 additional online resources:-

 1- WHO’s response to COVID-19  in the Eastern Mediterranean Region  Independent review by Dalberg Advisors February 2023 https://cdn.who.int/media/docs/default-source/evaluation-office/who-s-response-to-covid-19-in-the-emr---independent-review_february-2023_final.pdf?sfvrsn=130ab01a_3&download=true

 2- Distributional Impacts of COVID-19 in the Middle East and North Africa Region: https://www.worldbank.org/en/region/mena/publication/distributional-impacts-of-covid-19-in-the-middle-east-and-north-africa-region#:~:text=The%20report's%20findings%20suggest%20a,and%20how%20many%20people%20work).

Is the review written in accessible language?

Yes

Are all factual statements correct and adequately supported by citations?

Yes

Are the conclusions drawn appropriate in the context of the current research literature?

Yes

Is the topic of the review discussed comprehensively in the context of the current literature?

Yes

Reviewer Expertise:

Evidence-Based Healthcare, Clinical Practice Guidelines, Pediatrics and Child healthcare, Healthcare Informatics, Healthcare Quality and Safety, Healthcare services research, Improvement research, and Implementation research.

I confirm that I have read this submission and believe that I have an appropriate level of expertise to confirm that it is of an acceptable scientific standard, however I have significant reservations, as outlined above.

## References

[1] : Mapping of health innovations in response to the COVID-19 pandemic in Eastern Mediterranean and selected Arab Countries. *East Mediterr Health J* .2022;28(2) : 10.26719/emhj.22.028 130-143 10.26719/emhj.22.028 35304910

[2] : Hesitancy toward vaccination against COVID-19: A scoping review of prevalence and associated factors in the Arab world. *Hum Vaccin Immunother* .2023;19(2) : 10.1080/21645515.2023.2245720 2245720 10.1080/21645515.2023.2245720 37594508 PMC10443971

[3] : Mental health impact of the first wave of COVID-19 pandemic on healthcare workers in 12 Arab countries. *East Mediterr Health J* .2022;28(10) : 10.26719/emhj.22.065 707-718 10.26719/emhj.22.065 36382725

[4] : COVID-19 pandemic and economic impacts in Arab countries: Challenges and policies. *Research in Globalization* .2022;5: 10.1016/j.resglo.2022.100103 10.1016/j.resglo.2022.100103

# Reviewer report: Reviewer response for version 1

**DOI:** 10.5256/f1000research.156102.r233650

**Published:** 2024-05-11

**Authors:** Ahmad A. Alrasheedi

**Affiliations:** 1 Department of Family and Community Medicine, College of Medicine, Qassim University, Buraydah, Al Qassim Region, Saudi Arabia

Overall, this study is excellent and worth accepting as the authors evaluated the spread of the COVID-19 pandemic in a critical region, the Arab world, over three years (2020-2022). Understanding the epidemiology of COVID-19 in the Arab world and comparing it to the world could help researchers and decision-makers explore the best ways to deal with COVID-19 and may provide lessons for a more effective response to public health emergencies in the future. However, there are some suggestions to improve the article.

   

 1- **Type of the study**: Is this a review article or an original research? Based on the article's methodology and structure, this article is considered original (research article). The authors extracted primary/secondary data (raw data) from online websites and then analyzed and interpreted the data. In contrast, a review article or paper is based on other published articles. Review articles generally summarize the existing literature on a topic in an attempt to explain the current state of understanding of the topic. Therefore, this is not a review article because the authors did not base their methodology on a review of previous studies/research. The authors should reconsider this part!

 2- Suppose this article is considered an original study, which it appears to be. In that case, it is preferable to change the word “ **review**” in the title to another word, for example, “study,” so that the title is: “COVID-19 in the Arab countries: A three-year study” or “COVID-19 in the Arab countries: A study after three years”.

 3- In the "conclusions" section of the Abstract, Confirmed what? Confirmed cases or confirmed deaths. A word is missing!

 4- In the first line of the "Introduction" section, which coronavirus is? It is the novel coronavirus, or as it is named, SARS-CoV-2. In contrast, the disease caused by this virus is called COVID-19. I suggest it be written as "… declared coronavirus disease 2019 (COVID-19) as …".

 5- In the second sentence of the "Introduction" section, which years? 2020-2022 or 2021-2023; please specify!

 6- The reference No. 5 is old (2004). Is there any recent one? 

 7- In paragraph 4 of the "Introduction" section, please remove the phrase "the coronavirus." To be "… many different variants of SARS-CoV-2, including …". Also, in this statement: as long as **the coronavirus ** SARS-CoV-2 remains.

  Please remove the phrase "the coronavirus".

 8- In the "Methods" section, please provide the link/reference for each website if possible (Worldometer...).

 9- The exclusion criterion was **unofficial information regarding COVID-19 in all Arab countries**,... This phrase ( **unofficial information regarding COVID-19 in all Arab countries**) should be removed because it is known from the inclusion criteria (exclusion criteria are not the opposite of inclusion criteria).

 10- The following information was collected from each Arab country: total population, median age, number of monthly **confirmed,**... Confirmed what? Please make it clear.

 11- "Results" section: All Arab countries utilized real-time polymerase chain reaction (RT-PCR) as the testing method for SARS-CoV-2. I suggest to transfer this sentence to the "Introduction" section.

 12- UAE (19,632,329) recorded then the highest number of tests per million population (PMP), followed by Bahrain (5,960,320), and then Oman (4,695,724).

 Rephrasing the sentence to be clear, I suggest, " **The** UAE recorded the highest number of tests per million population (PMP) with 19,632,329, followed by Bahrain (5,960,320), and then Oman (4,695,724)." Always add "the" before UAE, USA, and UK while mentioned in the text.   

 13- The people in Qatar and the **United Arab Emirates** ... have

  [أع1] Use the abbreviation: the UAE.

 14- Table 1: Please identify the abbreviations in the table (Add footnotes), even if they have already been mentioned in the text.

 Please check the population number of Mauritania. 

 15- Table 2: define the abbreviations in the table (Add footnotes): UEA, SA, and PMP, as well as for other tables. Also, in column 10 (Q1-22), some numbers are not uniform in the way they are written.

   

 16- In the "Discussion" section, New Year season 

 New Year season? Do you mean influenza season, where URTIs, including coronaviruses, increase?

 17- Another reason for the spike increase is the slow uptake of the vaccine in **many countries.**

 Which countries? of the world, including Arab countries or in many Arab countries?

 18- During the first, second, and third quarters of 2020, the pandemic was under control in most Arab countries **due to** ... 

   Using the probability form, such as "mostly due to" or "probably because," is better.

 19- The first quarter of 2022 was a shockwave as it was recorded with the highest-ever number of COVID-19 confirmed cases with 3,235,665 peaked by Jordan with a total number of 630,811.

 To be clearer, I suggest you rephrase it to be: "During the first quarter of 2022, which was a shockwave, the Arab countries recorded the highest number of confirmed COVID-19 cases ever reported between 2020 and 2022. The total number of cases was 3,235,665, with Jordan reporting the highest number of cases at 630,811."

 20- Qatar was reported with the highest number of confirmed cases of COVID-19 in the fourth quarter of 2022

  Suggesting to rephrase by adding among Arab countries.

 21- In paragraph no. 12, ...Australia, **USA**, and Turkey have performed tests more than their populations.

  DEFINE USA: please write **the United States of America**. 

 22- In the "Strengths and limitations" section, However, there are a few limitations in our **review**.

  study or review? This will depend on your classification. 

 23- Although the number of **confirmed**, death, and subsequently recovered cases of...

 Confirmed what? Please make it clear.

 Thank you for your valuable study.

Is the review written in accessible language?

Yes

Are all factual statements correct and adequately supported by citations?

Yes

Are the conclusions drawn appropriate in the context of the current research literature?

Yes

Is the topic of the review discussed comprehensively in the context of the current literature?

Yes

Reviewer Expertise:

Family Medicine, Public Health, Common Health Problems (such as DM, HTN, Dyslipidemia, and Depression), and Epidemiology/Prevention of Infectious diseases such as COVID-19 and Influenza.

I confirm that I have read this submission and believe that I have an appropriate level of expertise to confirm that it is of an acceptable scientific standard, however I have significant reservations, as outlined above.

# Reviewer report: Reviewer response for version 1

**DOI:** 10.5256/f1000research.156102.r247533

**Published:** 2024-04-01

**Authors:** Muhammad Nauman Zahid

**Affiliations:** 1 College of Science, University of Bahrain, Sakhir, Southern Governorate, Bahrain

The authors have made an interesting attempt at “COVID-19 in the Arab countries: Three-year review.” The manuscript is interesting; however, the authors need to justify the scientific writing of the manuscript. Some of the general comments are provided below:

  

1. Considering the usage of several sources, what steps were taken to address any potential contradictions or inconsistencies discovered in the data collected?
2. In order to maintain the dataset's integrity, how were possible duplicate items found and dealt with?
3. What variables could account for the variations in COVID-19 cases and fatalities that were noted during the three years from January 2020 to December 2022? Could these tendencies have been impacted by any significant policy changes or events?
4. Taking into account the possible influence of variables including climate, public health initiatives, and alterations in population behavior, how were seasonal fluctuations in COVID-19 incidence and death taken into account in the analysis?
5. Could you provide further details about any possible biases or confounding factors, such as variations in healthcare access, population demographics, or government response tactics, that would affect how these cross-regional comparisons are interpreted?
6. According to the manuscript, vaccination campaigns were a major factor in the decline of COVID-19 cases, fatalities, and hospital admissions in Arab nations. Can you offer more proof or analysis to back up this claim, such as comparisons of COVID-19 results before and after vaccination campaigns or research on the efficacy of vaccines?
7. How were public opinions of vaccine safety and efficacy, vaccine hesitancy, and disinformation addressed during the vaccination campaign discussion? Were any tactics put in place to enhance vaccination uptake and remove obstacles to access?

Is the review written in accessible language?

Yes

Are all factual statements correct and adequately supported by citations?

Yes

Are the conclusions drawn appropriate in the context of the current research literature?

Yes

Is the topic of the review discussed comprehensively in the context of the current literature?

Yes

Reviewer Expertise:

Virologist

I confirm that I have read this submission and believe that I have an appropriate level of expertise to confirm that it is of an acceptable scientific standard, however I have significant reservations, as outlined above.

# Response

**Published:** 2024-04-21

**Authors:** Nasar Alwahaibi

**Affiliations:** Biomedical Science, Sultan Qaboos University, Muscat, Oman

**We would like to take this opportunity to express our thanks to the reviewer for the positive feedback and helpful comments.**

**Below are our responses, point-by-point to the queries of the reviewer.**

 The authors have made an interesting attempt at “COVID-19 in the Arab countries: Three-year review.” The manuscript is interesting; however, the authors need to justify the scientific writing of the manuscript. Some of the general comments are provided below:

  

1. Considering the usage of several sources, what steps were taken to address any potential contradictions or inconsistencies discovered in the data collected?

**Response**

**We use the Ministry of Health official websites in each Arab country as an initial source as they provide daily data, then verified with the WHO and Worldometer sources. In case of different data, which is not common, we referred to the Ministry of Health in each Arab country**.

  

1. In order to maintain the dataset's integrity, how were possible duplicate items found and dealt with?

**Response**

**The data are not duplicated, but sometimes there are differences between the WHO and Worldometer sources as they are reported at different times on the Ministry of Health's official websites.**

  

1. What variables could account for the variations in COVID-19 cases and fatalities that were noted during the three years from January 2020 to December 2022? Could these tendencies have been impacted by any significant policy changes or events?

**Response**

**The variables or factors that could account for the variations in COVID-19 cases and fatalities are mentioned in the discussion section and these include lockdown measures, mitigation strategies (such as implementing social distancing), employment and scale of diagnostic testing and vaccine intake, timely response of governments, weak healthcare system (physical resources and healthcare personnel and under reporting) and hence, all of these could be attributed to the “healthcare policy” of each country. Other variables include the occurrence of virus-related factors such as the fast-spreading Omicron variant, the UK variant, and the Alpha variant. Population or demographic characteristics such as age was also identified as a potential factor that may have influenced infection and death rates. We demonstrate in the discussion section that the ability of “healthcare systems to respond promptly against the COVID-19” such as implementing strict lockdown measures and mitigation strategies significantly reduced confirmed cases and fatalities. We also show in the discussion that countries that employed heavy testing protocols revealed higher number of cases and that under-reporting could have played a role in countries that did not employ large-scale testing.**

  

1. Taking into account the possible influence of variables including climate, public health initiatives, and alterations in population behavior, how were seasonal fluctuations in COVID-19 incidence and death taken into account in the analysis?

**Response**

**According to the data of COVID-19 fluctuations, the spikes of Covid-19 were connected to the spread waves but not the climate. For example, in Tunisia, the peaked deaths were in the third quarter of 2021 which is considered as hot climate whereas in Lebanon the peaked deaths were in the first quarter of 2021 which is considered as a cold climate. According to the public health initiatives (e.g. vaccination) and positive alteration in population behaviors, we mentioned in the paper how these two factors affected the decrease in the death’s numbers.**

  

1. Could you provide further details about any possible biases or confounding factors, such as variations in healthcare access, population demographics, or government response tactics, that would affect how these cross-regional comparisons are interpreted?

**Response**

**This is very good question, however, population demographics such as gender and age are not reported. Thus, we have added the following sentence as a limitation:**

**The lack of gender and age data in numerous Arab countries prevented us from conducting thorough comparisons and assessing potential risk factors.**

**Regarding the government response tactics, we have already mentioned in the discussion section that: During the first, second, and third quarters of 2020, the pandemic was under control in most Arab countries due to the implementation of extreme precautionary measures. The major measures include land, sea, and air route closure, nighttime or all-day curfew and lockdown, school and universities closure, worship places closure, prohibition of gatherings, closure of shops, malls, beaches, public parks, and gardens, cancellation of all cultural and sports events, festivals, seminars, and scientific meetings, and suspension of work in all government and private sectors.**

**Regarding the access to healthcare facilities during COVID-19 crises, this information is not available in all Arab countries. We know that GCC countries offered free medical treatment and vaccinations to all residents but this information is missing in other Arab countries.**

  

1. According to the manuscript, vaccination campaigns were a major factor in the decline of COVID-19 cases, fatalities, and hospital admissions in Arab nations. Can you offer more proof or analysis to back up this claim, such as comparisons of COVID-19 results before and after vaccination campaigns or research on the efficacy of vaccines?

**Response**

**Our results did not study the comparison of COVID-19 before and after vaccination campaigns. As suggested, the following paragraph has been added in the discussion section:**

**Research conducted in the United Arab Emirates regarding the inactivated BBIBP-CorV (Sinopharm) vaccine revealed that its efficacy against severe COVID-19 outcomes was 80% for hospitalization, 92% for critical care admission, and 97% for preventing death. ^46^ In addition, a study conducted in Morocco on the long-term efficacy of the inactivated BBIBP-CorV vaccine revealed a decrease in effectiveness, dropping from 88% to 64% six months after vaccination. ^47^ Furthermore, in Qatar, a different study demonstrated that the efficacy of BBIBP-CorV vaccine against SARS-CoV-2 infections decreased gradually, with a more rapid decline observed after the fourth month. This decline resulted in about 20% protection at five to seven months following vaccination. However, the vaccine's efficacy remained nearly 96% effective in preventing hospitalization and death six months after vaccination. ^48^**

  

1. How were public opinions of vaccine safety and efficacy, vaccine hesitancy, and disinformation addressed during the vaccination campaign discussion? Were any tactics put in place to enhance vaccination uptake and remove obstacles to access?

**Response**

**The following sentence has been added in the discussion section:**

**Certain tactics were used to enhance public awareness and acceptance of vaccine during these campaigns. One of the most effective tactics was to address public opinions of vaccine safety and efficacy by disseminating accurate information through authorized channels. This information was in different languages to reach out all in the community. Community engagement and healthcare guidance were also helpful. During the vaccination process, the uptake of vaccine was enhanced by removing any obstacle that might delay such a process. For example, setting up vaccination centers in different locations with an easy access and quick appointment. These centers had big area to accommodate more people at each time. Community outreach existed for those who could not go to these centers. ^44^**
